# Reactive Martini: Chemical Reactions in Coarse-Grained Molecular Dynamics Simulations

**DOI:** 10.1021/acs.jctc.2c01186

**Published:** 2023-06-16

**Authors:** Selim Sami, Siewert J. Marrink

**Affiliations:** Groningen Biomolecular Sciences and Biotechnology Institute, University of Groningen, Nijenborgh 7, 9747 AG Groningen, The Netherlands

## Abstract

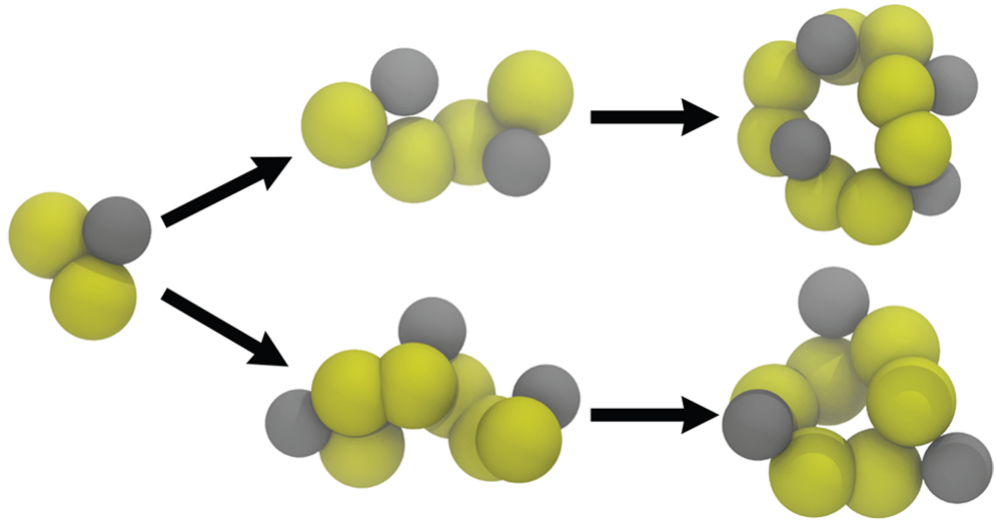

Chemical reactions are ubiquitous in both materials and the biophysical sciences. While coarse-grained (CG) molecular dynamics simulations are often needed to study the spatiotemporal scales present in these fields, chemical reactivity has not been explored thoroughly in CG models. In this work, a new approach to model chemical reactivity is presented for the widely used Martini CG Martini model. Employing tabulated potentials with a single extra particle for the angle dependence, the model provides a generic framework for capturing bonded topology changes using nonbonded interactions. As a first example application, the reactive model is used to study the macrocycle formation of benzene-1,3-dithiol molecules through the formation of disulfide bonds. We show that starting from monomers, macrocycles with sizes in agreement with experimental results are obtained using reactive Martini. Overall, our reactive Martini framework is general and can be easily extended to other systems. All of the required scripts and tutorials to explain its use are provided online.

## Introduction

Molecular simulations offer crucial insight into the dynamics of biomolecular processes and the design of novel technological materials at a temporal and spatial resolution unparalleled by experimental methods. A plethora of computational methods have been developed over the years in an attempt to balance two opposing requirements: computational cost of the simulations, which allows simulating larger systems for longer times, on the one hand, versus the complexity of the model, which is correlated to its ability to treat chemical phenomena accurately, on the other hand. Meeting both of these requirements has proven to be particularly challenging in the modeling of chemical reactions.

Ab initio (AI) molecular dynamics (MD) simulations, which treat electrons quantum mechanically, can account for chemical reactions inherently^1,2^ but are computationally too demanding to study most biologically or technologically relevant phenomena. Reactive models in classical all-atom (AA) MD simulations have been well developed^3−7^ and can be applied to significantly larger systems than AIMD, but are still limited in the spatiotemporal scales that can be covered.

In these cases, coarse grained (CG) approaches, where multiple atoms are grouped into a single bead, are necessary to alleviate the computational burden.^8^ However, reactive models in CGMD simulations have not received the same level of attention and development as AI or AA models, most likely as it appears a bridge too far to capture the reshuffling of electronic degrees of freedom with models that do not even follow the motion of the individual nuclei. It is clear that in such an approach, one needs to give up on modeling detailed and accurate reaction mechanisms and replace it with a more pragmatic approach. A common approach is to change the topologies of molecules that underwent a reaction in some predefined intervals and based on certain criteria (distance, angles, energies). This can be done either statically by starting or stopping the simulation or dynamically by tracking the reaction progress with a lambda parameter.^9^ These approaches has proven successful in capturing reactions with dissipative particle dynamics (DPD)^10,11^ as well as CGMD.^12−15^ However, the intermittent evaluations severely limit the speed of the simulation, in particular for systems where many such reactions can take place. Moreover, unless costly energy calculations are included, the effect of the environment on the reactivity is not properly accounted for. Another method has recently been pioneered by the group of Voth, called reactive coarse-grained (RCG) dynamics.^16^ In RCG, an empirical valence-bond-like approach is used to couple the products and reactants of the reaction, modeled at the CG level. The CG interactions are fine-tuned to reproduce the reaction potential of mean force obtained from an all-atom description in a bottom-up multiscale framework. One thus obtains a CG model capable of undergoing reactions with realistic energy barriers. A drawback of RCG, however, is the requirement of dedicated software to perform multistate simulations, which severely limits the availability of the approach.

Here, we opt for a different, more pragmatic approach using the Martini model,^17^ which is one of the most employed CG models, recently improved with the 3.0 version.^18^ It has been successfully applied to a variety of fields ranging from biophysics to materials science.^19,20^ Some extensions to the Martini have also been developed in order to account for various chemical phenomena not treated by the standard Martini model such as the polarizable water model,^21^ Go-potentials to stabilize secondary structures of proteins,^22^ and most recently two models that can form and break chemical bonds: the titratable Martini model for constant pH simulations,^23,24^ where protons can reversibly bind to water and other titratable sites, and the sticky Martini model,^25^ where silica beads can form and break bonds, allowing to study its polymerization.

All these extensions have mostly relied on the creative use of virtual sites and dummy particles (dummy particles have a mass and take part in the dynamics while not representing any actual atom, and virtual sites are constructed by other particles and not take part in the dynamics) combined with Lennard-Jones interaction sites on these particles. In the case of titratable Martini, a single attractive dummy particle, which reversibly binds to the proton bead, has been combined with a single repulsive dummy particle that modulates the angle of binding. In the case of sticky Martini, four attractive dummy particles have been combined with four repulsive virtual sites in a stellated octahedral configuration to match the bonding geometry of silica beads. While these repulsive sites have been shown to successfully modulate the angle of binding, it has not been explored yet how it affects the nonbonded interactions in nonbinding angles, i.e., whether the reactive beads still behave as standard Martini beads in nonbinding angles that can be seamlessly combined with the library of standard Martini bead types. Another challenge that has so far not been explored is the binding dihedral angle: Both in titratable and sticky Martini, the reactive moieties are molecules or atoms consisting of a single Martini bead. Consequently neither the parametrization of the binding dihedral angle through nonbonded potentials nor the study of multibead reactive molecules has so far been explored. This remains an essential step in extending reactive CG models to larger systems.

One of many systems that chemical reactions play an important role is the supramolecular assembly of self-replicating macrocycles, which are believed to have been crucial in the origin of life.^26−31^ A commonly used monomer building block for artificial self-replication consists of a benzene-1,3-dithiol and a peptide chain. The benzene-1,3-dithiol allows for the creation of macrocycles through the formation of two disulfide bonds. Without the peptide chain, initially 2mer chains are formed, which then lead to 3mer and 4mer macrocycles. With the peptide chain, specifically one that can form β sheets, these 3mers and 4mers, which are called the precursors, then convert into hexamer or heptamer macrocycles, which self-assemble through stacking and hydrogen bonding interactions and induce fiber growth.^26^ Modeling of the self-replication process proves to be extremely challenging as it requires treatment of extremely large systems for a very long time while successfully modeling the disulfide bond formation chemical reaction and the self-assembly of the macrocycles through β sheet formation. Preformed fibers have been previously studied with the Martini model,^30,31^ however, the macrocycle formation has not been possible with the standard Martini model.

In this paper, a generic and pragmatic approach for making reactive Martini models is presented. First, the parametrization strategy and the validation of the model are described in detail. The addition of reactive particles which interact through a tabulated potential allows us to retain standard Martini interactions in nonreactive directions (e.g., π–π stacking), unlike the sticky-Martini approach. In addition, the tabulated potentials enable the inclusion of energetic barriers that can be used to tune the reaction rate. Next, an extension to treat reactivity of multibead molecules is presented, which required a new strategy for the parametrization of dihedrals through the use of nonbonded potentials. Finally, reactive Martini is applied to study the macrocycle formation using the benzene-1,3- dithiol molecule. It is shown that a system initially consisting of only monomers evolves into a system of macrocycles of mostly 3mers and 4mers, in line with experimental results.^26^ The reactive model presented here can be adapted and applied to different systems and molecules quite straightforwardly through the use of scripts provided in the Supporting Information.

## Results and Discussion

The main challenges and our design goals for a reactive Martini framework are (1) being generic and applicable to a variety of systems; (2) being able to capture bond formation and breaking, but not the reaction mechanisms, at the CG level at desired time scales; (3) modulating the bond, angle, and dihedrals between reactive molecules with nonbonded potentials, as bonded potentials cannot be used for this purpose; (4) while modulating these terms with nonbonded potentials, ensuring that both reacted and nonreacted species retain standard Martini interactions in nonreactive angles (e.g., π–π stacking).

With these goals in mind, first a simpler case, i.e., the bond formation between single beads, is explored, which only requires dealing with reactive bonds and angles. Then, the extension to multibead molecules is described, which also involves both proper and improper dihedrals. Finally, the model is showcased with an application, where the macrocycle formation is treated with the reactive model.

### Reactive Model: Bonds and Angles

The basis for the reactive model is the creation of an angle-dependent potential through the use of tabulated potentials. To obtain the reactive potential (*V*_reactive_) at the required bonding angle, a virtual site (VS_R_) is placed on the direction of the reactive bond that is separated by a distance *d* with a tabulated potential of
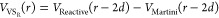
1between VS_R_ particles. Here, *V*_Martini_ is the standard potential for the corresponding Martini beads, which retains its parameters. In this way, the reactive bead will interact near the reactive angles with the potential *V*_Reactive_ and at other angles with the standard Martini interactions (*V*_Martini_). The distance *d* between the central bead and VS_R_ determines the width of the angle dependence, and it can be tuned accordingly. Thus, a single extra particle is sufficient for the creation of the angle dependence without any repulsive sites. The functional form and different components of *V*_Reactive_ are explained and illustrated (Figures S1–2) in detail in the Supporting Information. The reactive bond length is parametrized to match the AA solvent-accessible surface area (Figure S4).

**Figure 1:**
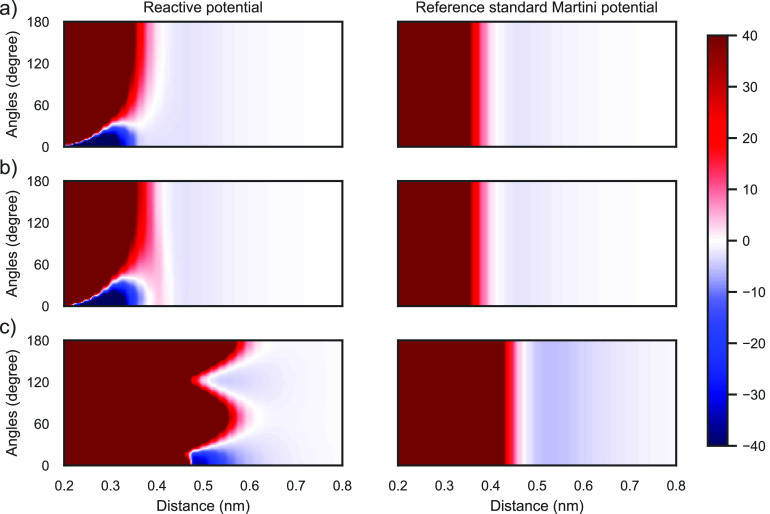
Interaction energy (kJ/mol) between two reactive sites as a function of the distance and angle. Angle is given as the difference to the reactive minimum, i.e., 0° are the respective equilibrium angles. Left panel contains the reactive potentials while the right one contains the corresponding reference standard Martini potentials for: (a) reactive potential from this work, (b) reactive potential from this work with an additional reaction barrier, and (c) reactive potential by Carvalho et al.^25^ and the corresponding reference potential.^32^ Potentials above 40 and below −40 kJ/mol are colored the same as these energies.

The results of this approach are seen in Figure 1a. Within the 60° range of the equilibrium angle (0°), it has the reactive potential and at larger angles, it behaves exactly the same as the reference standard Martini potential. It is also possible to add reaction barriers with the proposal model, which can be important to modulate the reaction rate. This is shown in Figure 1b, where a reaction barrier is added between the Martini minimum and the reactive bond minimum. Finally, in Figure 1c, the consequences of using repulsive sites are presented for the reactive bead parametrized by Carvalho et al.:^25^ While the reactive angle behaves correctly (i.e., there is a well at the reactive bond length), the potential is overly repulsive at angles where the repulsive sites reside (90°, 180°). While Carvalho et al. argue that self-interaction of these particles does not play a dominant role in the polymerization process they are studying, using this approach for a general reactive Martini would be challenging due to a mismatch with standard Martini interactions.

### Reactive Model: Proper and Improper Dihedrals

As discussed above, in the case of a reactive multibead molecule, additional considerations beyond the reaction angle are needed, such as the parametrization of the improper and proper dihedrals of the reaction product. The key idea is to use nonbonded interactions to capture these interactions, instead of bonded potentials which are used under standard (nonreactive) conditions. To illustrate the concept, the thiophenol molecule is used, which can react with another thiophenol by forming a disulfide bridge, resulting in diphenyl disulfide. Note that while we use thiophenol to illustrate the reactive Martini framework, by simply changing bond lengths, angles and depth of potentials in the scripts provided in the Supporting Information, the framework can also be used and validated for other molecules.

**Figure 2:**
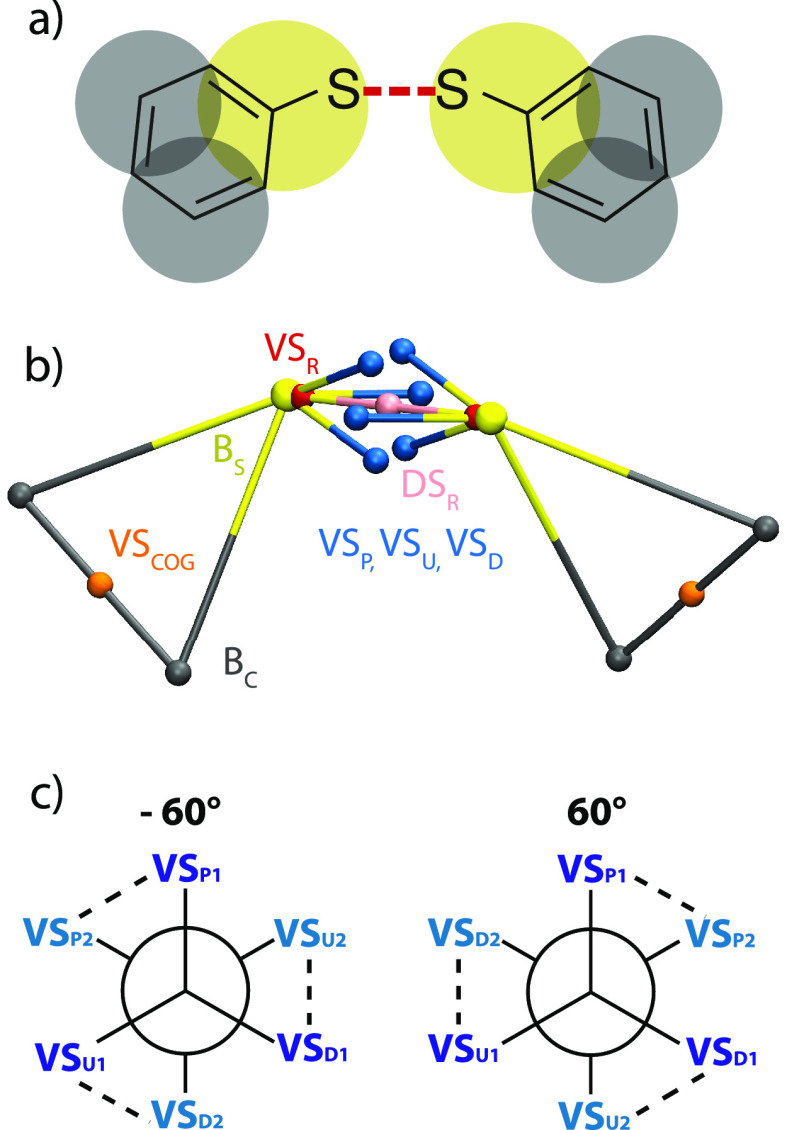
Setup for capturing bonds, angles, and dihedrals with nonbonded interactions. (a) AA and CG representation of the diphenyl disulfide molecule with the reactive bond shown in red dashes. (b) CG beads (B_C_, B_S_), virtual sites (VS_COG_, VS_R_, VS_P_, VS_U_, VS_D_), and dummy particles (DS_R_) used in the reactive model. (c) Schematic representation of the two staggered configurations of the virtual sites enforcing the dihedral angle. Subscripted numbers refer to the two molecules.

In Figure 2a, the atomistic structure and the corresponding Martini CG mapping are given for diphenyl disulfide, where sulfide-containing moieties are drawn in yellow, the remaining carbon-containing beads in dark gray, and the reactive bond in red dashes. Matchingly, yellow (B_S_) and dark gray (B_C_) beads in Figure 2b represent these CG beads, which retain their standard Martini nonbonded interactions (SC6 and TC5, respectively). The orange particle (VS_COG_) is a virtual site placed between the two B_C_ beads, as commonly done in connected ring systems in Martini^33^ to define angle and dihedral terms and has no nonbonded interactions. The red bead (VS_R_) denotes the reactive center as discussed earlier, which interacts with other VS_R_ beads with the *V*_VS_R__ potential from eq 1.

While for many reactions the reactive bond will form on the plane of the reactive bead and its neighbors, this is not always the case. For example, in thiophenol, the reaction occurs above and below the plane of the rings, which in standard force fields would have been handled by an improper dihedral angle potential. In the reactive model, with the single reactive virtual site (VS_R_), only one of these configurations could be satisfied, since it cannot move above and below the plane. While using a dummy site instead of a virtual site for the reactive particle would alleviate this issue, as it would then take part in the dynamics to move above and below the plane, the reactive particle must have a fixed distance (not constrained) to the central bead for stability reasons due to the large attractive (*V*_reactive_) and repulsive (*V*_Martini_) forces that must be canceled out precisely at bonding distances. To solve this issue, VS_R_ is connected to pink dummy site DS_R_, which can move above and below the plane freely. In this way, the bond length of VS_R_ to B_S_ remains fixed while it is free to move above and below the plane of the ring. The DS_R_ bead does not have any nonbonded interactions, and in Figure 2b, the DS_R_ beads of the two reactant molecules overlap with each other.

**Figure 3:**
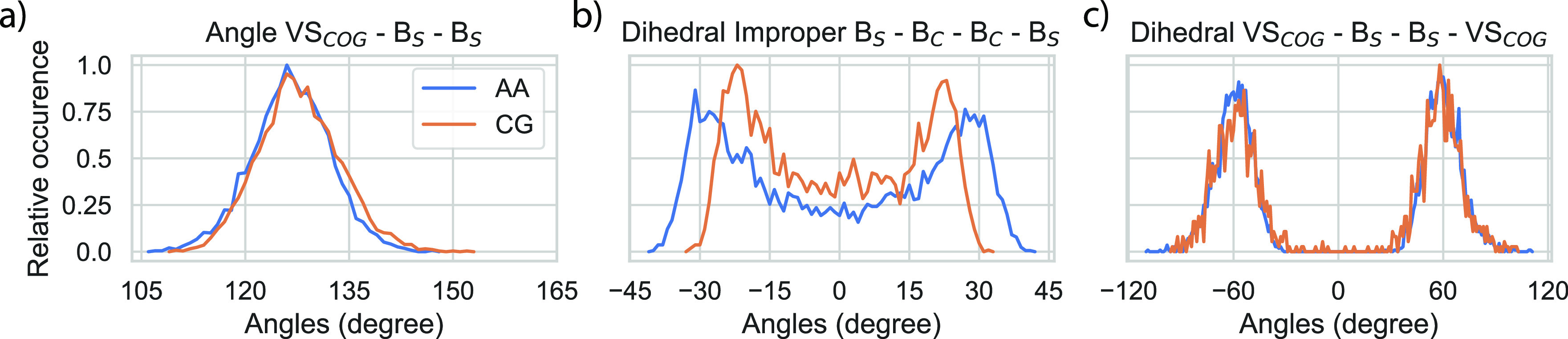
Validation of matching angle and dihedral distributions for diphenyl disulfide. AA and CG distributions of: (a) angles, (b) improper dihedrals, and (c) proper dihedrals. Constituting particle names are given in the titles and are visualized in Figure 2b. Each panel is normalized to have its maximum equal to one.

The final consideration is the proper dihedral angle centered at the reactive beads. To enforce this, additional virtual sites are used to lock the reacting molecules into the correct dihedral angles. In thiophenol, this is the proper dihedral angle between the phenyl rings. This dihedral has populations centered at −60° and 60° (see Figure 3c), meaning that the two rings are tilted this much with respect to each other. Three additional virtual sites (blue) are added to create this configuration: one in the plane of the ring (VS_P_), positioned 25° away from the center of the reactive bond, and two that are with the same length and angle but are out of plane by 120° (VS_U_) and −120° (VS_D_) with labels referring to “up” and “down”. Then, these three virtual sites of each molecule are forced together in a staggered configuration at −60 and 60°, while disfavoring the 180° configuration (Figure 2c). This is done with a tabulated nonbonded interaction using a raised-cosine-shaped function (see Figure S3 for the shape) between the two in-plane and the two pairs of −120° and 120° virtual sites. The strength of this interaction can be modulated to match the dihedral profile, even for asymmetric profiles, by giving different interactions strengths to different pairs. For changing the minimum energy angle(s), the position or number of the virtual sites can be changed. For example, if the dihedral profile had two populations at −90° and 90°, two virtual sites would be placed instead of three in the plane of the molecule, similarly positioned at an angle from the center of the reactive bond.

Next, angle and dihedral distributions achieved by the model described above are matched and compared to the AA reference. In Figure 3, distributions are shown for atomistic simulations of the reacted molecule (diphenyl disulfide) mapped into CG beads (blue) and for CG simulations with the reactive model (orange). The angle-dependent potential described earlier successfully captured the AA angle distributions for the VS_COG_-B_S_-B_S_ angle (Figure 3a). Similarly, addition of the DS_R_ particle allows capture of the out-of-plane bending successfully (Figure 3b). Finally, the three VS_D_ particles added for modulating the proper dihedrals are also shown to work in Figure 3c for the VS_COG_-B_S_-B_S_-VS_COG_ dihedral.

As a final validation of the angle dependence shown in Figure 1 and of the claim that the reactive bead behaves as a standard Martini bead in nonreactive directions, the mass density and the radial distribution function (RDF) between the B_S_ particles were computed for the reacted molecule diphenyl disulfide with standard and reactive Martini. Mass densities were in perfect agreement with each other (1263 g/L) for the standard and reactive Martini and also within 10% error with respect to the experiments (1353 g/L),^34^ which is typically the accepted range for Martini models. Additionally, RDF profiles of the distance between the B_S_ particles, shown in Figure S5, are in very good agreement for the standard and reactive Martini models, validating that the reactive site behaves as a standard Martini bead in nonreactive directions. Snapshots from the formation of a B_S_-B_S_ bond with these angle and dihedral configurations are shown in Figure S6.

One of the main benefits in simulation time in CG models comes from the higher time step. Because of the steepness of Lennard-Jones interactions with small sigma parameters, previous reactive models (titratable and sticky Martini) had to lower the time step to 5–8 fs from the commonly used 10−20 fs in Martini simulations. In this work, because smooth tabulated potentials were used and repulsive sites were avoided, it was possible to retain a 10 fs time step. On the other hand, currently, in GROMACS, tabulated potentials are implemented only with the group neighbor scheme, which is significantly slower. However, this is only an algorithmic problem, and work is underway to implement tabulated potentials with the Verlet scheme in GROMACS.

We expect the addition of many extra dummy and virtual sites to have minimal effect on the overall computational cost because: (i) In most applications, reactive particles will be a small percentage of the particles in the simulation box. Consequently, the overall simulation will still benefit from the speed improvements of the Martini model without being strongly affected by the extra particles of several reactive sites. (ii) There is only 1 extra particle (the dummy site) that takes part in the dynamics, and the rest are virtual sites that are constructed from the positions of other particles. The effect of virtual sites on the simulation time is significantly less and these are commonly used in many Martini models. (iii) Extra virtual sites only interact among themselves, i.e., VS_R_ only interacts with other VS_R_ particles, and V_SP/U/D_ also only interacts with other VS_P/U/D_ particles. Consequently, assuming that it is not a system full of these particles, the additional extra interactions that need to be computed will be quite minimal.

### Study of Macrocycle Formation

**Figure 4:**
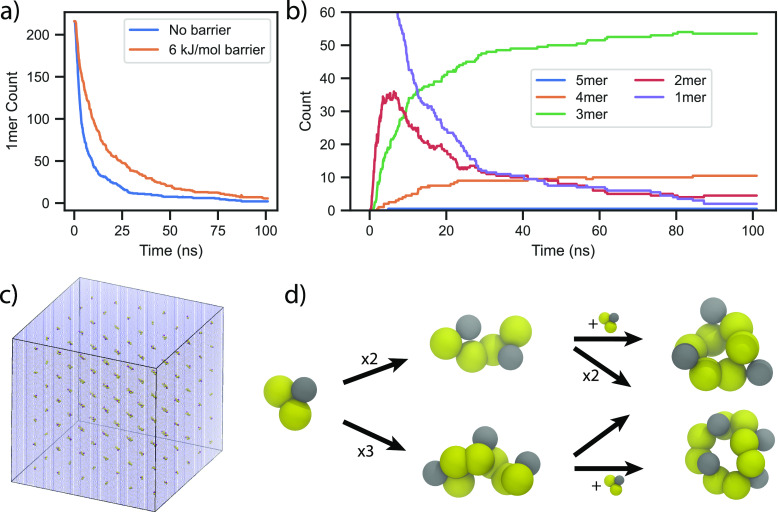
Ring formation of benzene-1,3-dithiol with reactive Martini. (a) 1mer count as the simulation progresses for the models with and without the reaction barrier. (b) Chain/ring count of different sizes as the simulation progresses for the model without the reaction barrier. (c) Starting simulation box consisting of 216 1mers. (d) Snapshots of rings/chains of different sizes from the simulation and the conversion flow between them.

The thiophenol molecule that was used for parametrization has only a single reactive site, i.e., the sulfur containing bead. While this is a good proof-of-principle system, in order to form macrocycles, two such sites are needed per molecule. Consequently, macrocycle formation is studied on the benzene-1,3-dithiol molecule, where two of the parametrized reactive sites are used. A system of 216 benzene-1,3-dithiol monomers is solvated in water (Figure 4c) at a concentration of 1.33 mM, in line with experiments.^26^ Then, the macrocycle size is studied over time, where the results are averaged over two simulations. In Figure 4a, the monomer count is shown for the two reactive potentials described earlier: one without a reaction barrier and one with a 6 kJ/mol barrier. It can be seen that the monomers are consumed over time but more slowly so for the model with the reaction barrier. This shows that the reaction rate can be modulated with the use of a barrier in the reactive potential. It should be noted that the intention is not to match experimental reaction rates, which takes days and would be impossible to simulate, but instead to get the relative time scales in the system correct. One such relation with regard to fiber formation would be the reaction rate against the β sheet formation rate.

Formation of larger chains and rings, as monomers are consumed, is shown in Figure 4b for the model without the barrier. First, 2mer concentration is maximized after 5 ns, but these 2mers start being consumed to form larger rings, and after 100 ns, very few 2mers are left in the system. 2mer chains are used to create 3mers and 4mers in high concentrations; about 50 3mer and 10 4mers are created by the end of the 100 ns simulation (Figure 4d). These results are in agreement with experiments, where without the peptide chain, benzene-1,3-dithiol system mostly forms 3mers and 4mers. Only a single 5mer was produced in the two simulations that the results are averaged over, which we consider being below the detection range of experiments. The reason why larger rings do not occur easily is that 1mers form 2mer chains and 2mers form either 3mer or 4mer chains, both of which can react with itself to form rings and avoid growing further.

## Conclusions

In this work, a novel approach to model chemical reactivity within the Martini framework is demonstrated. Using a single extra particle with a tabulated potential, the angle dependence of the reactive bond was achieved while alleviating important issues with previous CG reactive models. Additionally, proper and improper CG dihedrals were parametrized for the first time through the use of short-range nonbonded potentials. The model then was applied to the study of macrocycle formation of the benzene-1,3-dithiol molecule through the formation of disulfide bonds and showed that ring sizes in agreement with experimental results are obtained.

Overall, this approach presents a straightforward way of parametrizing new reactive CG Martini models using the scripts provided in the Supporting Information. The virtual site placements, which can be typically difficult to manage, are done automatically based on bond, angle information provided by the user. With the implementation of the Verlet scheme for tabulated potentials, 10 fs time step will allow these reactive simulations to be performed at a minimal additional cost compared to standard Martini simulations. We also expect the more specialized cases such as multiple reactive sites per bead^25^ to work out-of-the-box by having multiple VS_R_ particles at the correct positions, which would make multiple reactive angles.

Finally, the angle-dependent nonbonded potential described in this work could be used for various other applications, such as to stabilize hydrogen bonds in Martini to handle different folding structures of proteins (α helix vs β sheet), to fine tune DNA/RNA hybridization and to further improve the titratable model for constant pH simulations. Stabilization of β sheets will be particularly useful for studying the fiber formation of the ring systems in this work, with the additional peptide chains included.

## Methods

### Force Field Parametrization

AA diphenyl disulfide was parametrized using the Q-Force protocol,^35^ which uses quantum mechanical calculations to automatically derive AA force field parameters. Default settings were used, meaning that CM5 charges were combined with OPLS Lennard-Jones parameters.^36^ CG thiophenol parameters were taken from Alessandri et al.^33^ A standard CG Martini model was parametrized for diphenyl disulfide by matching bonded parameters to the Q-Force-based AA bonded parameters. Parameterization of the reactive model is discussed in the Results and Discussion section in detail. All force field parameters can be found in the Supporting Information.

### Run Parameters

GROMACS 2018.8 was used,^37^ as this is the last version that supports the group cutoff scheme, which is currently needed for tabulated potentials. The group cutoff scheme was combined with a neighbor list size of 1.2 nm, which was updated every 10 steps. Nonbonded interactions were cutoff at 1.1 nm, and these interactions were shifted to zero at the cutoff. A velocity rescale thermostat^38^ with 1 ps coupling time was used to keep the temperature at 298 K. A Parrinello–Rahman barostat^39^ with 12 ps coupling time was used to keep the pressure at 1 bar. For the contraint solver, LINCS was used with an order of 8 and 2 iterations. A time step of 10 fs was used, and this was stable for all studied systems.

## Supporting Information Available

Reactive model details and functional forms, additional figures (PDF)
Force field files, run parameters, relevant scripts (ZIP)
