# Supplementary material for: Reactive Martini: Chemical Reactions in Coarse-Grained Molecular Dynamics Simulations
Source: J Chem Theory Comput. 2023 Jun 16;19(13):4040–6. doi: 10.1021/acs.jctc.2c01186 (PMC10339680; doi:10.1021/acs.jctc.2c01186)
Supplement: Supplementary file 1 — ct2c01186_si_001.pdf [file ct2c01186_si_001.pdf]

Supporting Information: Reactive Martini: Chemical Reactions in coarse-grained molecular dynamics simulations

Selim Sami*,†,‡ and Siewert J. Marrink*,†

†*Groningen Biomolecular Sciences and Biotechnology Institute, University of Groningen, Nijenborgh 7, 9747 AG Groningen, The Netherlands*

‡*Present address: Kenneth S. Pitzer Theory Center and Department of Chemistry, University of California, Berkeley, Berkeley, California 94720, United States*

E-mail: s.sami@berkeley.edu; s.j.marrink@rug.nl

Components and the functional form of the reactive potential

The reactive potential is a combination of 4 potentials:

1) The standard Martini Lennard-Jones potential at distances equal to or greater than the equilibrium distance R_{min} of the standard bead type:

$$V_{\text{martini}}(r) = \begin{cases} 4\epsilon\left[\left(\frac{\sigma}{r}\right)^{12} - \left(\frac{\sigma}{r}\right)^{6}\right], & \text{if } r \geq R_{\text{min}} \\ V_{\text{martini}}(R_{\text{min}}), & \text{otherwise} \end{cases} \tag{S1}$$

In this work, the standard Martini bead type of the reactive particle is SC6, which has a σ and ε value of 0.41 nm and 2.6 kJ/mol, respectively, which corresponds to a potential minimum (R_{min}) of 0.46 nm ($\sigma\sqrt{2}$). The resulting potential is shown in Fig. S1a.

2) The reaction barrier, which is given by a Gaussian function:

$$V_{\text{barrier}}(r) = V_{\text{height}} \times exp\left(-\frac{(r - R_{\text{barrier}})^2}{2w_{\text{barrier}}^2}\right) \tag{S2}$$

This potential is only used for the reactive model with the reaction barrier and the height of the barrier(V_{height}), its peak position (R_{barrier}), and its width (w_{barrier}) are set to 6 kJ/mol, 0.4 nm and 0.02 nm, respectively. The resulting potential is shown in Fig. S1b.

3) The well for the reactive bond, which is given by a Gaussian function at distances greater or equal to the reactive bond length (R_{BB}):

$$V_{\text{well}}(r) = \begin{cases} -V_{\text{depth}} \times exp\left(-\frac{(r-R_{\text{BB}})^2}{2w_{\text{well}}^2}\right), & \text{if } r \geq R_{\text{BB}} \\ V_{\text{well}}(R_{\text{BB}}), & \text{otherwise} \end{cases} \tag{S3}$$

The reactive bond length (R_{BB}), well depth (V_{depth}), and the width of the well (w_{well}) are set to 0.28 nm, 65 kJ/mol and 0.033 nm, respectively. The resulting potential is shown in Fig. S1c.

4) A harmonic potential for the reactive bond at distances shorter than the reactive bond length (R_{BB}):

$$V_{\text{harmonic}}(r) = \begin{cases} \frac{k_{\text{harmonic}}}{2}(r - R_{\text{BB}})^2, & \text{if } r \leq R_{\text{BB}} \\ 0, & \text{otherwise} \end{cases} \tag{S4}$$

The harmonic force constant (k_{harmonic}) is set to 250000 kJ/mol/nm^2. The resulting potential is shown in Fig. S1d.

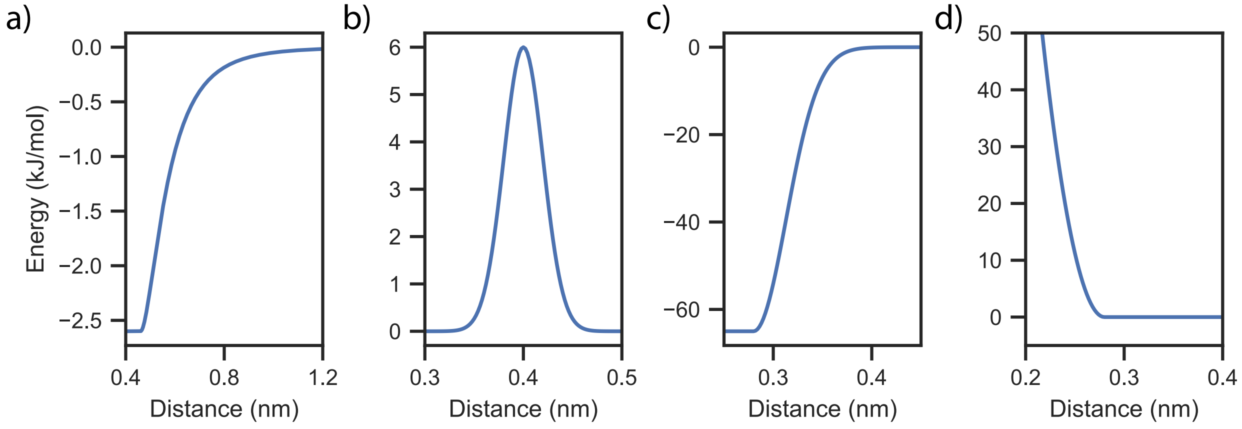

Figure S1: Components of the reactive potential ($\text{V}_{\text{Reactive}}(r)$) used this work. a) The standard Martini Lennard-Jones potential at distances equal to or larger than equilibrium distance R_{min}; b) the reaction barrier; c) the well for the reactive bond; d) the harmonic potential for the reactive bond at distances shorter than the reactive bond length (R_{BB}).

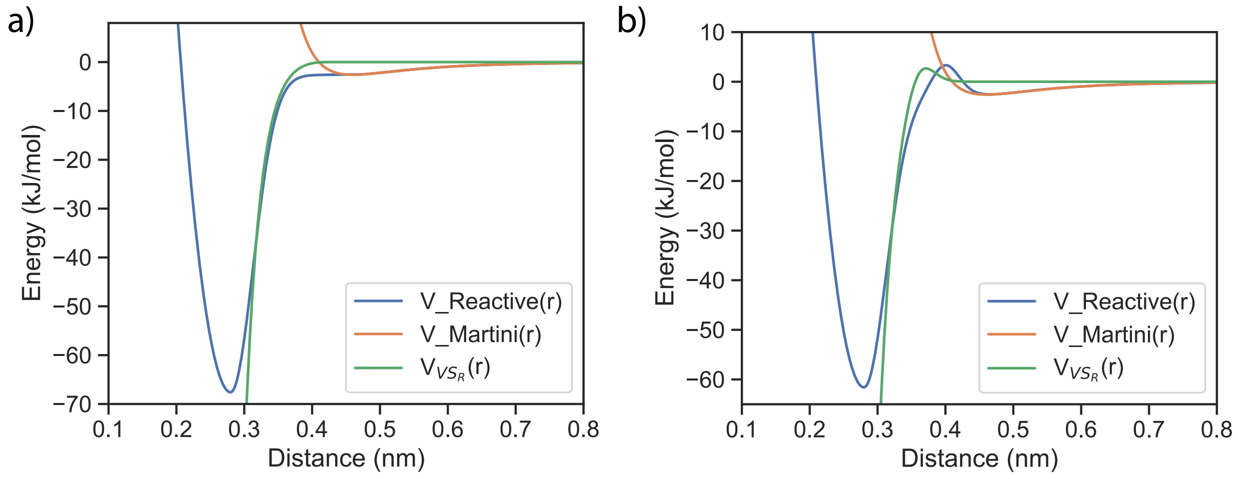

Figure S2: $\text{V}_{\text{VS}_\text{R}}(r)$, $\text{V}_{\text{Reactive}}(r)$ and $\text{V}_{\text{Martini}(r)}$ potentials shown in eq. 1 for the cases without (a) and with (b) reaction barrier.

The sum of the potentials above leads to the final reactive potentials shown in Fig. S2

for the cases with and without reaction barriers.

The functional form of the dihedral potential

For modulating the dihedral angle, a raised-cosine function is used as discussed in the main manuscript. The functional form of this potential is:

$$V_{\text{dihed}}(r) = \begin{cases} -\frac{V_{\text{dihed}}}{2} * (1 + cos(\frac{\pi}{w_{\text{dihed}}(r - R_{\text{DD}})}), & \text{if } R_{\text{DD}} - w_{\text{dihed}} \leq r \leq R_{\text{DD}} + w_{\text{dihed}} \\ 0, & \text{otherwise} \end{cases} \tag{S5}$$

The depth of the barrier (V_{dihed}) and its width (w_{dihed}) are parameterized as 8 kJ/mol and 0.06 nm, respectively. R_{DD} is the equilibrium distance between the matching pairs of VS_D, VS_U and VS_P particles. This distance is computed (not parameterized) based the reactive bond length (R_{BB}) and the DS_R-B_S-$\text{VS}_{\text{D,U,P}}$ angle described below.

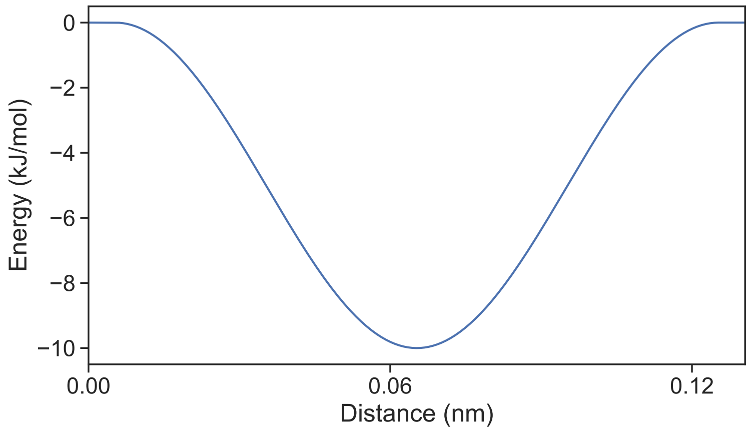

Figure S3: Shape of the raised cosine function used for modulating the dihedrals.

Remaining parameters of the reactive model

Besides the 9 parameters (V_{height}, R_{barrier}, w_{barrier}, R_{BB}, V_{depth}, w_{well}, k_{harmonic}, V_{dihed}, w_{dihed}) discussed above (6 if no reactive barrier is used) that determines the shape of potentials,

here we restate the remaining four that determines the positions of virtual sites.

1) The distance d between the central bead (B_S) and the reactive virtual site VS_R, discussed in the main article as the distance *d*. This determines the width of the angle dependence, i.e., how quickly the potential switches from the reactive to the standard Martini potential, and it can be tuned accordingly. This is parametrized as 0.02 nm in this work.

2) The angle and the force constant of the VS_{COG}-B_S-DS_R angle, which determines the equilibrium reactive angle. These are parametrized to be 128 degrees and 600 kJ/mol/nm^2, respectively.

3) The DS_R-B_S-$VS_{D,U,P}$ angle, which determines in-plane (VS_P) and off-plane ($VS_{D,U}$) angles for the virtual sites responsible for the dihedral profile. Too small angles would result in a very short R_{DD}, which would require a very small time step for accurate treatment of the dihedrals. Too large angles would result in a very long R_{DD}, which could start interfering with the rest of the model. We found that for this model 25 degrees gives a good balance, ensuring both stability at 10 fs timestep and that the dihedral potential vanishes around 0.12 nm (see Fig. S3).

Parametrizing the reactive bond length

Unlike AA force fields that typically match quantum mechanical (QM) bond lengths, CG bond lengths are adjusted more freely and they do not have to match QM or AA values. What is more important for the Martini model is to match the volume of the CG model to the AA model, therefore bond lengths are typically adjusted as needed to match the AA volume, which is characterized by the solvent accessible surface area (SASA). Similarly, we have chosen the S-S bond length to get a good match to the AA SASA (Figure S4).

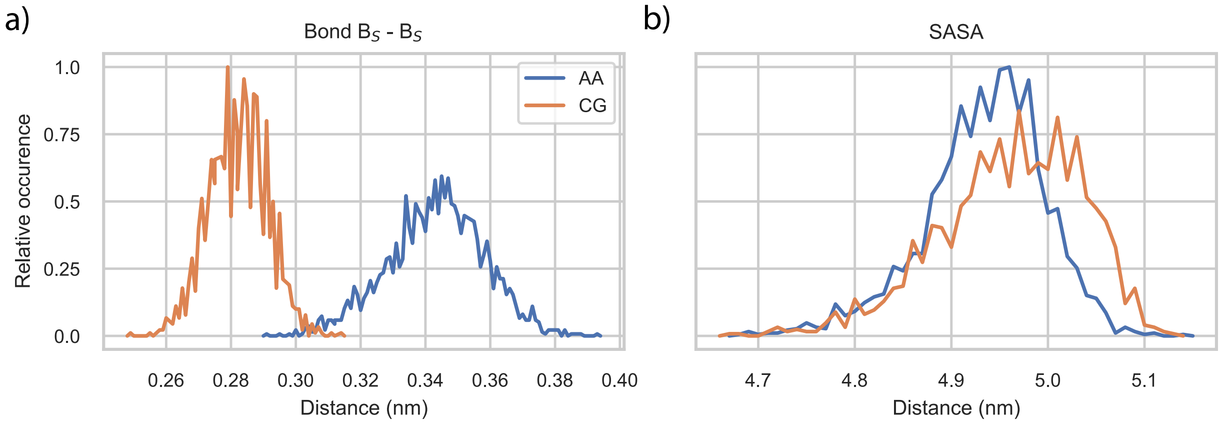

Figure S4: CG and AA distributions of the a) reactive bond length and b) the solvent-accessible surface area.

Additional figures

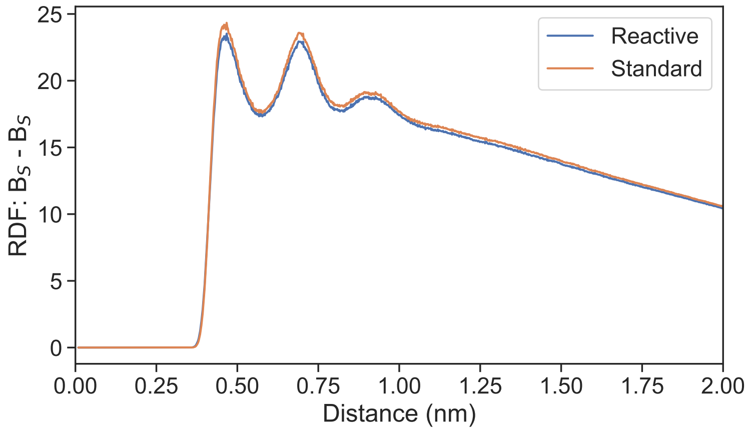

Figure S5: Radial distribution function between B_S beads that are not bonded to each other for the reactive and standard Martini models.

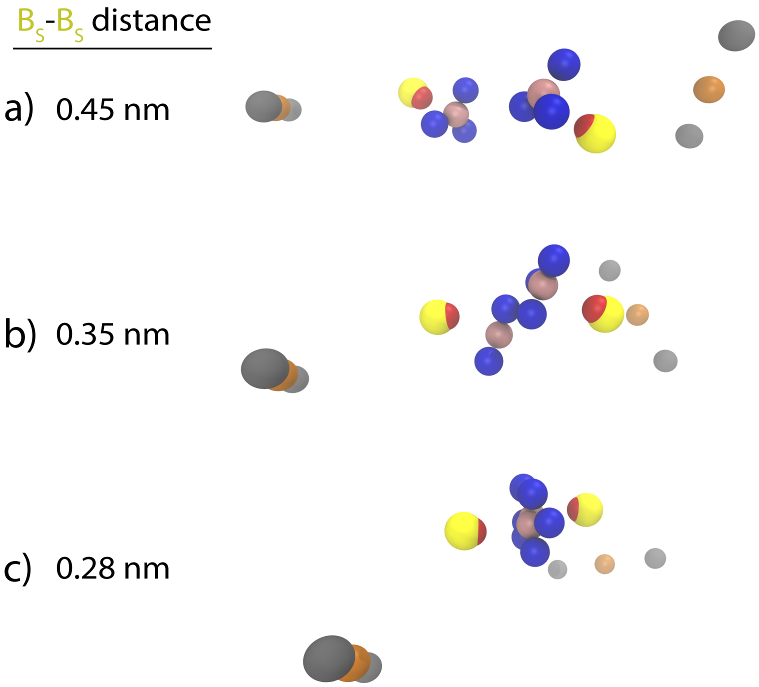

Figure S6: Snapshots from the formation of a B_S-B_S bond. a) at 0.45 nm, standard Martini Lennard-Jones distance; b) 0.35 nm, mid-point of the reaction; c) 0.28 nm, bonded distance.
